# Nutraceutical Effects of Lycopene in Experimental Varicocele: An “In Vivo” Model to Study Male Infertility

**DOI:** 10.3390/nu12051536

**Published:** 2020-05-25

**Authors:** Pietro Antonuccio, Antonio Micali, Domenico Puzzolo, Carmelo Romeo, Giovanna Vermiglio, Violetta Squadrito, Jose Freni, Giovanni Pallio, Vincenzo Trichilo, Maria Righi, Natasha Irrera, Domenica Altavilla, Francesco Squadrito, Herbert R. Marini, Letteria Minutoli

**Affiliations:** 1 Department of Human Pathology of Adult and Childhood, University of Messina, 98125 Messina, Italy; pietro.antonuccio@unime.it (P.A.); romeo.carmelo@unime.it (C.R.); 2 Department of Biomedical and Dental Sciences and Morphofunctional Imaging, University of Messina, 98125 Messina, Italy; amicali@unime.it (A.M.); puzzolo@unime.it (D.P.); giovanna.vermiglio1@unime.it (G.V.); freni.jose.89@gmail.com (J.F.); maria.righi@unime.it (M.R.); daltavilla@unime.it (D.A.); 3 Department of Clinical and Experimental Medicine, University of Messina, 98125 Messina, Italy; violettasquadrito@gmail.com (V.S.); gpallio@unime.it (G.P.); vincenzo.trichilo@unime.it (V.T.); nirrera@unime.it (N.I.); hrmarini@unime.it (H.R.M.); lminutoli@unime.it (L.M.)

**Keywords:** varicocele, testis, carotenoids, lycopene, diet, nutraceuticals, oxidative stress, apoptosis, rat

## Abstract

Varicocele is one of the main causes of infertility in men. Oxidative stress and consequently apoptosis activation contribute to varicocele pathogenesis, worsening its prognosis. Natural products, such as lycopene, showed antioxidant and anti-inflammatory effects in several experimental models, also in testes. In this study we investigated lycopene effects in an experimental model of varicocele. Male rats (*n* = 14) underwent sham operations and were administered with vehicle (*n* = 7) or with lycopene (*n* = 7; 1 mg/kg i.p., daily). Another group of animals (*n* = 14) underwent surgical varicocele. After 28 days, the sham and 7 varicocele animals were euthanized, and both operated and contralateral testes were weighted and processed. The remaining rats were treated with lycopene (1 mg/kg i.p., daily) for 30 days. Varicocele rats showed reduced testosterone levels, testes weight, Bcl-2 mRNA expression, changes in testes structure and increased malondialdehyde levels and BAX gene expression. TUNEL (Terminal Deoxynucleotidyl Transferase dUTP Nick End Labeling) assay showed an increased number of apoptotic cells. Treatment with lycopene significantly increased testosterone levels, testes weight, and Bcl-2 mRNA expression, improved tubular structure and decreased malondialdehyde levels, BAX mRNA expression and TUNEL-positive cells. The present results show that lycopene exerts beneficial effects in testes, and suggest that supplementation with the tomato-derived carotenoid might be considered a novel nutraceutical strategy for the treatment of varicocele and male infertility.

## 1. Introduction

Varicocele is one of the main causes of infertility in men, and it represents an important clinical problem worldwide [1]. Varicocele is characterized by an abnormal dilatation of the veins in the pampiniform plexus within the scrotum, and in 90% of patients it is mainly present on the left side [2]. Molecular processes, such as the release of pro-apoptotic molecules which activates apoptosis, and the accumulation of pro-inflammatory cytokines which exacerbates inflammation, contribute to the pathogenesis of varicocele [1,3].

Nowadays, the exact mechanisms that correlate varicocele and infertility are still unknown; however, scrotal hyperthermia, hypoxia and oxidative stress seem to play important roles [4]. The treatment of varicocele and the care of the associated fertility problems still represent an area of interest for researchers, although many advances have occurred in recent years.

Antioxidants use is considered among the proposed methods as the most appropriate therapeutic approach to reduce the effects of varicocele, such as infertility [5].

Recently, experimental studies demonstrated that functional foods of natural origin have antioxidant, anti-inflammatory and anti-apoptotic effects in varicocele [1,3,6,7,8,9]. The principal antioxidants present in the Mediterranean diet (MD) are carotenoids; in particular β-carotene, α-carotene, lycopene, β-cryptoxanthine, lutein and zeaxanthin account for over 90% of all carotenoids [10]. Among these, lycopene represents between 21% and 43% of total carotenoids contained in the blood [11]. Among the vegetables, tomatoes are a main component of MD and, in addition to lycopene, they contain significant quantities of vitamins C and E, folate, polyphenols, and other carotenoids such as phytoene and phytofluene [12]. Significant concentrations of lycopene are only found in a select number of foods (such as tomato, watermelon, guava and pink grapefruit) and lycopene intake coming from fresh and processed tomato products is highly relevant [13].

Many epidemiological studies have suggested that intake of lycopene-containing foods, as well as blood lycopene concentrations, are inversely related to the incidence of cardiovascular disease and prostate cancer [14]. Lycopene may provide some of the cardiovascular or cancer protection associated with tomato intake, but is not likely to be the only bioactive compound in tomatoes. In this regard, many researchers have done relevant work to better understand the role of lycopene and its derivatives in the process of chronic diseases [15]. For example, the characterization and study of β-carotene 9′,10′-oxygenase (BCO2) showed that this enzyme can catalyze the excentric cleavage of both provitamin and non-provitamin A carotenoids to form apo-10′-carotenoids, including apo-10′-lycopenoids, from lycopene [16,17], underlining the crucial impact of liver enzymatic function on carotenoid metabolism, including lycopene and lycopenoids.

Once ingested, lycopene is absorbed into the intestinal mucosa via passive diffusion, incorporated into dietary lipid micelles and chylomicrons, and transported to various organs. Different factors may influence lycopene absorption, such as age, gender, hormones, smoking, alcohol, and the interaction with other components of the diet [18]. From a translational point of view, it is particularly interesting to consider that lycopene obtained from processed and heated tomato is better absorbed [19], and lycopene distribution in tissues depends on its structure. The highest concentrations have been found in the testes, adrenal glands, liver and prostate, thanks to the high concentrations of polyunsaturated fatty acids [10,20].

Thanks to its high concentration in testes, lycopene may be useful for the treatment of the diseases that affects the male reproductive tract. However, conflicting results have been so far reported: it has been demonstrated that lycopene restores spermatogenesis, modulating oxidative stress and apoptosis [21,22], but contrasting data described the negative role of lycopene in experimental testicular ischemia/reperfusion, thus demonstrating that a long-term treatment with lycopene might be not effective in restoring testicular damages [23].

As far as we know, no data are currently available on the effects of lycopene in varicocele. Moreover, while there has been great interest in the antioxidant properties of lycopene, other mechanisms of actions that may or may not be related to antioxidant function could be implicated in this medical condition. Of course, multiple factors, including carotenoid metabolism, molecular biological properties, and their interaction with genetic and epigenetic factors, must undoubtedly be carefully considered to better define the role and application of carotenoids and their metabolites in human health and disease [15]. Therefore, the aim of this study was to evaluate the effects of lycopene in an experimental rat model of varicocele, eventually providing further insight into the mechanisms of action, and to investigate whether this nutraceutical approach might preserve testis structure.

## 2. Materials and Methods

### 2.1. Animals and Experimental Procedures

A total of 28 male Sprague-Dawley rats aged 7 weeks and weighing 200–230 g were purchased from Charles River Laboratories Italia srl (Calco, Italy). Animals were maintained under controlled environmental conditions, with a cycle of 12 h light/dark and a temperature of approximately 23 °C, and provided with food and water ad libitum. The standards for care and use of animals as stated in the ARRIVE (Animal Research: Reporting In Vivo Experiments) guidelines were followed in the present study and all procedures were approved by the Italian Ministry of Health (authorization number 90/2017 - PR). All animals were anesthetized with an intraperitoneal (i.p.) injection of ketamine and xylazine (75/2.5 mg/kg, i.p., respectively). Varicocele was induced in fourteen animals, as previously described in detail [24,25]. A group of male rats (*n* = 14) underwent a sham operation to evaluate the possible response of the testis to the surgical inflammatory stress and were administered with vehicle (corn oil; *n* = 7) or with lycopene (*n* = 7; 1 mg/kg i.p., daily) throughout the experimental period. The i.p. route of administration was chosen as it would overcome the possible malabsorption by different food formulations experimentally administered in rats, on the basis of the animal model used, which aims to reproduce an acute experimental model of varicocele; the dose of lycopene (1 mg/kg) was chosen accordingly to previous studies [26].

After a period of 28 days following the surgical procedure, 7 varicocele animals and 7 sham rats were euthanized with an intraperitoneal (i.p.) injection of ketamine and xylazine (75/10 mg/kg, i.p., respectively) and blood and both operated and contralateral testes were weighted and processed for biochemical, histopathological and immunohistochemical evaluation. The remaining sham and varicocele rats were treated with lycopene (1 mg/kg i.p., daily) for 30 days and then euthanized to collect blood and both testes for the analysis.

### 2.2. Drugs

Lycopene was purchased from Sigma Aldrich, Milan, Italy (Cat. Number #36275). Lycopene has a purity ≥ 95.0%. All chemicals and reagents were commercially available reagent grades.

### 2.3. Determination of Testosterone

Testosterone was measured in serum by ELISA methodology using a commercially available kit, according to the protocol suggested by the manufacturer. In brief, blood was obtained from cardiac puncture and serum was achieved by centrifugation at 1000× *g* for 10 min. An HRP (Horseradish Peroxidase)-conjugate and the specific antibody were added, followed by substrates and stop solution. The mean absorbance was calculated using a microplate reader at 450 nm and correlated with the values of the standard curve. Data were expressed in ng/mL.

### 2.4. Malondialdehyde Assay

Malondialdehyde (MDA) levels in testes were measured to evaluate lipid peroxidation and oxidative stress [27]. Testes were weighed to obtain the same amount of tissue for each animal and were mixed with 1.15% of KCl solution to be homogenized using a homogenizer (Miccra Gmbh, Müllheim, Germany). The homogenate (0.1 mL) was added to a 0.2 mL of sodium dodecyl sulfate (SDS; 8.1%), 1.5 mL of acetic acid (20%), 1.5 mL of thiobarbituric acid (0.8%) and distilled water (700 mL). Samples were boiled at 95 °C for 1 h and then centrifuged at 3000× *g* for 10 min. The supernatant was collected and the absorbance was read at 650 nm with a spectrophotometer.

### 2.5. Real Time (RT) PCR Assay

Rat testes were collected to extract total RNA with Trizol LS reagent (Invitrogen, Carlsbad, CA, USA). A spectrophotometer (NanoDrop Lite, Thermo Fisher, Waltham, MA, USA) was used to quantify RNA and 2 μg of RNA were reverse transcribed with the Superscript VILO kit (Invitrogen). A final volume of 20 μL per well was obtained adding 1 μL of cDNA to the EvaGreen qPCR Master Mix (Biotium Inc., Fremont, CA, USA). Samples were run in duplicate, GADPH was used as housekeeping gene and the final concentration of primers was 10 μM. Target genes were BAX and Bcl-2.

Primers used for target and reference genes were:

GADPH

Fw: 5′CTCATGACCACAGTCCATGC3′

Rv: 5′TTCAGCTCTGGGATGACCTT′

BAX

Fw: 5′CGAGCTGATCAGAACCATCA3′

Rv: 5′CTCAGCCCATCTTCTTCCAG′

Βcl-2

Fw: 5′ATACCTGGGCCACAAGTGAG3′

Rv: 5′TGATTTGACCATTTGCCTGA3′

Results were expressed as 2-ΔΔCt, as n-fold increase of gene expression and compared to sham.

### 2.6. Histological Evaluation

Testes were immediately fixed in freshly prepared Bouin solution, dehydrated in graded ethanol, cleared in xylene and embedded in paraffin (Paraplast, Supplies SPI, West Chester, PA, USA). 5 μm sections were cut with a rotary microtome (RM2125 RT, Leica Instruments, Nussloch, Germany), cleared with xylene, rehydrated in graded ethanol, and stained with hematoxylin and eosin (HE). The slides were photographed with a Nikon Ci-L (Nikon Instruments, Tokio, Japan) light microscope; the images were taken with a digital camera Nikon Ds-Ri2 and processed to the final magnification of 800×.

### 2.7. Morphometric Evaluation

Five non-serial sections per animal were evaluated for each group. Two experienced investigators performed morphological evaluation independently, blinded to the experimental group of each animal. Five microscopic fields (MFs), all including two entire seminiferous tubules from 10 non-serial sections of each group, were considered. For morphological assessment, the mean tubular diameter (MTD) was calculated by measuring the diameters of 100 separate seminiferous tubules, all showing a circular profile. A Peak Scale Loupe 7x (GWJ Company, Hacienda Heights, La Quinta, CA, USA) micrometer was used as a scale calibration standard to calculate the diameters, expressed in μm. Seminiferous epithelium was evaluated with the Johnsen’s scoring system [28], as modified for rodents [29]. Briefly, a score of 10 to 1 was given to each tubule according to its epithelial organization: 10, full spermatogenesis; 9, many late spermatids and disorganized tubular epithelium; 8, few late spermatids; 7, no late spermatids, few early spermatids; 6, no late spermatids, arrest of spermatogenesis at the spermatid stage, disturbance of spermatid differentiation; 5, no spermatids, many spermatocytes; 4, no spermatids, few spermatocytes, arrest of spermatogenesis at the primary spermatocytes stage; 3, only spermatogonia; 2, no germ cells, Sertoli cells only; 1, no seminiferous epithelial cells, tubular sclerosis.

### 2.8. Measurement of Apoptosis with Terminal Deoxynucleotidyl Transferase dUTP Nick End Labeling (TUNEL) Assay

For the TUNEL technique, an apoptosis detection kit (In situ Apoptosis Detection kit, Abcam, Cambridge, UK) was used. From the same blocks used for histological evaluation, 5 μm sections were cleared in xylene and rehydrated in graded ethanol. After permeabilization with proteinase K, endogenous peroxidase activity was stopped with 3% H_2_O_2_ in methanol. Sections were incubated with terminal deoxynucleotidyl transferase, with biotin-labeled deoxynucleotides, with streptavidin–horseradish peroxidase conjugate and with the diaminobenzidine solution, and counterstained with Harris hematoxylin. The slides were photographed with a Nikon Ci-L (Nikon Instruments, Tokyo, Japan) light microscope using a digital camera Nikon Ds-Ri2. From each group, two trained observers without knowledge of the treatment blindly evaluated from 100 seminiferous tubules the distribution of apoptosis, expressed as percentage of tubules with apoptotic cells (%TWAC), and the mean number of TUNEL-positive cells per tubule, expressed as apoptotic index [30].

### 2.9. Statistical Analysis

The statistical significance of differences among groups was performed with ANOVA comparison tests. Mann–Whitney U tests with Bonferroni correction was used for the statistical analysis of histological scores. A *p* value ≤ 0.05 was considered statistically significant. Values are provided as mean ± standard deviation (SD).

## 3. Results

### 3.1. Lycopene Effects on Testis Weight

All testes were weighted after sacrifice. No significant differences were observed in testes weight among any sham groups: therefore, for the clarity of data, a single value is provided as representative of sham.

Varicocele operated testes showed a weight significantly lower than sham (−41%). On the contrary, in varicocele contralateral testes, and in both the operated and the contralateral testes of rats treated with lycopene, no significant differences were observed versus sham (+3%, −11% and +10%, respectively) (Table 1).

### 3.2. Lycopene Effects on Testosterone and MDA Levels

Testosterone levels were similar in sham groups; therefore, for the clarity of data, a single value is provided as representative of controls. A significant decrease in testosterone levels was observed in varicocele operated animals compared to sham (−55%), whereas lycopene administration caused a significant increase in testosterone levels (+50%) (Table 1).

MDA levels were markedly increased in varicocele rats compared to sham animals. Lycopene treatment significantly decreased lipid peroxidation, thus demonstrating the efficacy of lycopene in reducing oxidative stress (Table 2).

### 3.3. Lycopene Modulates the Expression of BAX and Bcl-2

**Figure 1:**
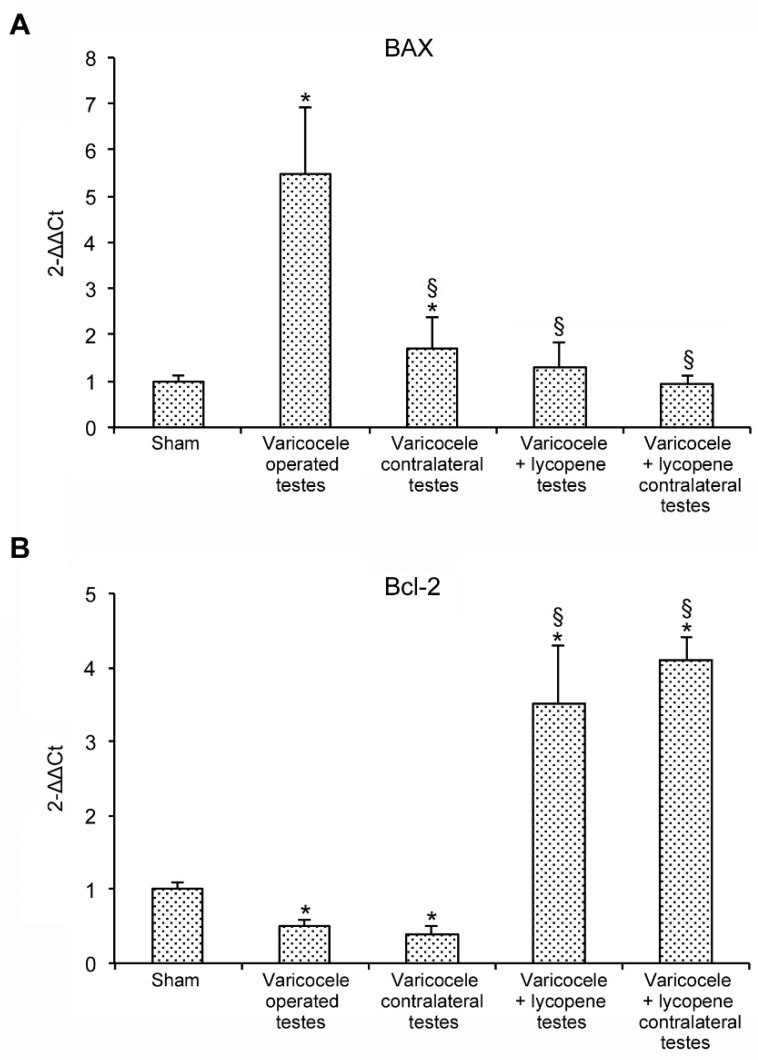
Real time PCR analysis for BAX (**A**) and Bcl-2 (**B**) in testes of sham and varicocele rats treated with vehicle or lycopene (1 mg/kg i.p., daily), respectively. * *p* < 0.05 versus sham rats; § *p* < 0.05 versus testes of varicocele-treated rats. Bars represent the mean ± SD of 7 experiments.

Animals subjected to varicocele showed an increased mRNA expression of the pro-apoptotic BAX compared to sham and also to contralateral testes. The treatment with lycopene significantly reduced BAX mRNA expression in both operated and contralateral testes, compared to the testes of varicocele rats (Figure 1A).

The anti-apoptotic Bcl-2 showed an opposite trend. In fact, Bcl-2 mRNA expression was significantly reduced in varicocele rats, whereas lycopene administration significantly increased its expression in both operated and contralateral testes, thus demonstrating that apoptotic pathway was reduced following lycopene treatment (Figure 1B).

### 3.4. Lycopene Administration Counteracts Seminiferous Tubules Damages

**Figure 2:**
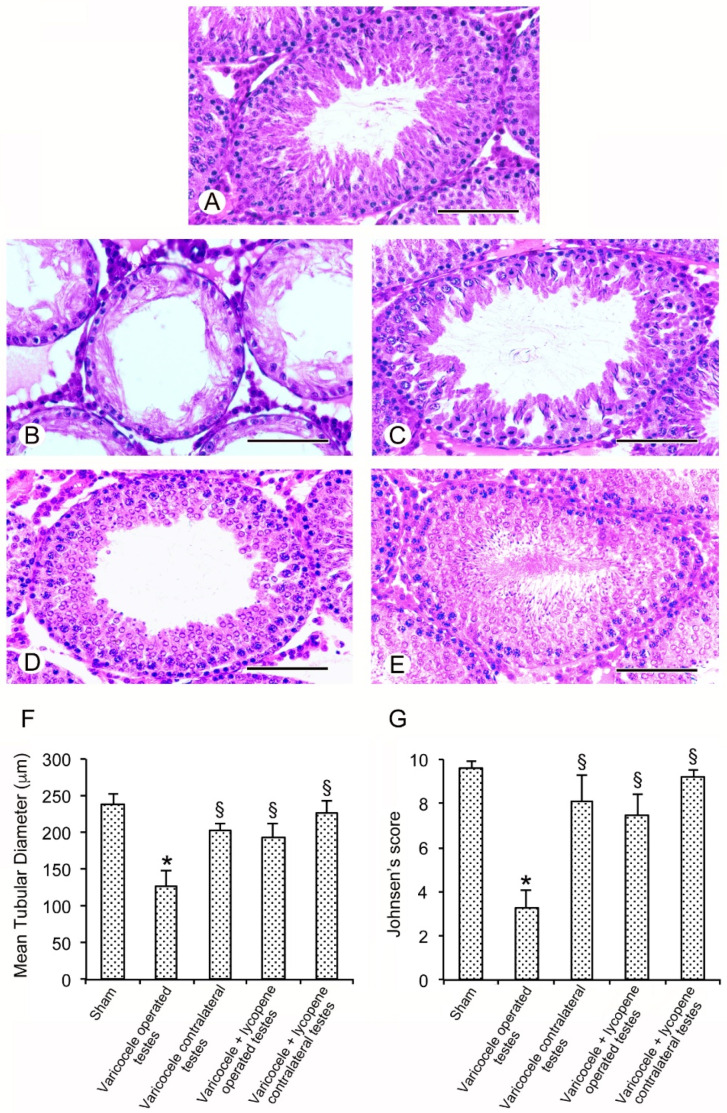
Histological organization of the testes with Hematoxylin-Eosin stain. (**A**): Sham rats. A normal tubular structure is present. (**B**): Varicocele operated rats. The seminiferous epithelium is thinner and sometimes atrophic with disorganized germ cells and residual sperm tails in the adluminal compartment. In the extratubular compartment, a marked edema is present. (**C**): Contralateral testes of varicocele operated rats. The seminiferous tubules show a better-preserved epithelium, with many spermatids and some immature spermatozoa. The extratubular compartment shows only a mild edema. (**D**): Varicocele rats treated with lycopene. The seminiferous epithelium is better preserved and shows round or elongated spermatids. A mild edema is present in the extratubular compartment. (**E**): Contralateral testes of varicocele rats treated with lycopene. The seminiferous epithelium has a normal structure with many spermatids and mature spermatozoa; also, the extratubular compartment is close to normal. **(F)**: Quantitative evaluation of the mean tubular diameter in the different groups of rats. (**G**): Johnsen’s score in the different groups of rats. * *p* < 0.05 versus sham; § *p* < 0.05 versus varicocele operated. (Scale bar: 50 µm).

All sham groups showed a normal morphology of both the seminiferous tubules and the extratubular compartment. Therefore, for the clarity of results, a single image is provided as representative of sham (Figure 2A) and a single datum is provided for the MTD and the Johnsen’s score (Figure 2F,G).

A destruction of the seminiferous epithelium was present in the testes of varicocele rats; a thin and sometimes atrophic epithelium, with disorganized germ cells and residual sperm tails in the adluminal compartment, was observed (Figure 2B). Consequently, the tubules showed a reduced mean diameter and the Johnsen’s score was low (Figure 2F,G). A marked edema was present in the extratubular compartment. The seminiferous tubules showed a better-preserved epithelium (Figure 2C) in the contralateral testes of varicocele rats, with many spermatids and some immature spermatozoa. The tubules had a larger mean diameter (Figure 2F) and the Johnsen’s score was higher than that of the sham group (Figure 2G). The extratubular compartment showed only a mild edema.

The seminiferous epithelium was better preserved and showed round or elongated spermatids in the testes of varicocele rats treated with lycopene (Figure 2D); the tubules had a significant large mean diameter (Figure 2F) and an increased Johnsen’s score (Figure 2G). Only a mild edema was observed in the extratubular compartment. The germinal epithelium had a normal structure, with many spermatids and mature spermatozoa in the contralateral testes of varicocele rats treated with lycopene; the extratubular compartment was also close to normal (Figure 2E). The seminiferous tubules had normal mean diameters (Figure 2F) and Johnsen’s score (Figure 2G).

### 3.5. Lycopene Administration Counteracts Seminiferous Epithelial Cells Apoptosis

**Figure 3:**
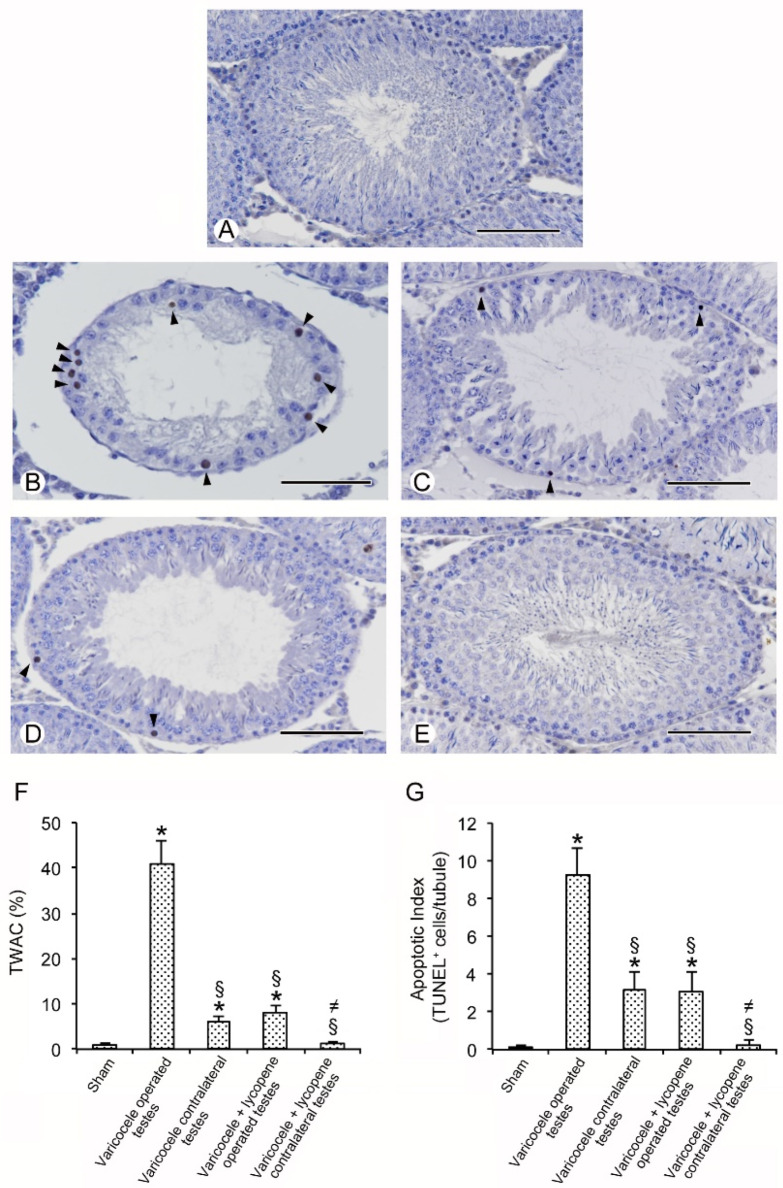
Assessment of apoptosis in the testes with TUNEL staining technique. (**A**): In sham rats no TUNEL-positive cells can be observed. (**B**): Varicocele operated rats. In the seminiferous epithelium a large number of TUNEL-positive germ cells (arrowheads) are present along the periphery of the tubules. (**C**): Contralateral testes of varicocele operated rats. Few isolated TUNEL-positive germ cells (arrowheads) are present at the periphery of the seminiferous tubules. (**D**): Varicocele rats treated with lycopene. Few peripheral TUNEL-positive cells are present in the seminiferous tubules. (**E**): Contralateral testes of varicocele rats treated with lycopene. Rare TUNEL-positive cells can be demonstrated. (**F**): Tubules with apoptotic cells (TWAC) (expressed in %) in the different groups of rats. (**G**): Apoptotic index (apoptotic cells/tubule) in the different groups of rats. * *p* < 0.05 versus controls; § *p* < 0.05 versus varicocele; ≠ *p* < 0.05 versus contralateral testes of varicocele operated rats and testes of varicocele rats treated with lycopene. (Scale bar: 50 µm).

All sham animals showed identical morphology following TUNEL assay. Therefore, for the clarity of results, a single image is provided as representative of the sham group (Figure 3A), and a single datum is provided for the TWAC and apoptotic index (Figure 3F,G). No TUNEL-positive germ cells were observed in the seminiferous tubules in the sham animals.

On the contrary, a large number of TUNEL-positive germ cells, evenly distributed along the periphery of the tubules, were observed in the seminiferous tubules of varicocele rats (Figure 3B). In fact, both the TWAC and apoptotic index were significantly higher than those observed in the sham group (Figure 3F,G). By contrast, few isolated TUNEL-positive germ cells were present at the periphery of the seminiferous tubules in the contralateral testes of varicocele rats (Figure 3C). Both the TWAC and apoptotic index were significantly lower than those of varicocele rats (Figure 3F,G).

Only few peripheral TUNEL-positive cells were present in the seminiferous tubules in the testes of varicocele rats treated with lycopene (Figure 3D); the TWAC and apoptotic index were significantly reduced (Figure 3F,G). Rare TUNEL-positive cells were present in the seminiferous tubules in the contralateral testes of varicocele rats treated with lycopene (Figure 3D), so that TWAC and apoptotic index were similar to those for the sham group (Figure 3F,G).

## 4. Discussion

Several therapeutic strategies have been proposed to reduce testicular damage and counteract human infertility related to varicocele. The use of functional foods of natural origin in recent decades has sparked the interest of researchers in the possible use of nutraceuticals for the treatment of varicocele. A previous study indicated that a high intake of green vegetables, fruits, whole grains, fish, chicken, and low-fat dairy products, and a simultaneous reduced consumption of meat, treated foods, sweets, and high-fat products improved the quality of semen [31].

The MD is a plant-based eating plan that ameliorates sperm concentration, total sperm count and sperm motility in men affected by reduced fertility [32]. Among the nutrients available in the MD, lycopene is a carotenoid mainly contained in tomatoes [33]. Lycopene is considered as a strong antioxidant and anti-apoptotic agent [34], but the role of lycopene as a possible antioxidant product is controversial because of its low distribution in tissues. Most of the effects of lycopene seem to be ascribed to lycopenoids, which are the active metabolic products resulting from lycopene metabolism.

According to some evidence, the positive anti-cancer effects on the prostate are related to the consumption of lycopene/lycopenoids-containing foods, such as tomatoes.

Moreover, tomatoes contain other bioactive molecules, such as vitamins C, vitamin E, folate, polyphenols, phytoene and phytofluene, which are other carotenoids in addition to lycopene; thus, the efficacy and the curative effects of lycopene could be related to the combination of all these components, and not only to lycopene [14].

In this regard, it has been shown that lycopenoids reduce the proliferation of cancer cells, induce apoptosis, regulate flow through the cell cycle, induce nuclear transcription factors, enhance cell-to-cell communication, and modulate androgen signaling [14]. Moreover, the consumption of lycopene-containing foods and the tissue specific expression of carotenoid cleavage enzymes determine tissue lycopenoid concentrations [14]. For example, enzymatic kinetic analysis indicates that the non-provitamin A carotenoids, including lycopene, are preferentially cleaved over provitamin A carotenoids, indicating a key role of β-carotene 9′,10′-oxygenase (BCO2) in non-provitamin A carotenoid metabolism. Accordingly, non-provitamin A carotenoids were shown to induce several phase II enzymes both in vivo and in vitro [35,36]. Then, because induction of phase II detoxifying or antioxidant genes by dietary carotenoids represents an important cellular defense in response to oxidative and electrophilic insults, more research is clearly needed to identify and characterize additional carotenoid metabolites and their biological activities.

So far, a clinical study, involving 47,365 persons, has demonstrated the reduced risk of prostate cancer following lycopene intake, and a reduction of male infertility and improvement of sperm quality using lycopene as a dietary supplement, thus increasing sperm number and viability [33,37]. In the light of these observations, in the present study we chose not to measure lycopene in testes, but we preferred to have an indirect read-out of its bioavailability and efficacy. Since it has been shown that the bioavailability of lycopene varied significantly depending on the administered matrix [38], we have chosen, in this experimental protocol, the i.p. route of administration, thus overcoming a possible malabsorption by different food formulations, as well as allowing us to reach an adequate blood and tissue bioavailability. As to its efficacy, we evaluated its antioxidant activity by testing the behavior of MDA.

Varicocele is a multifactorial disease, and oxidative stress seems to play an important role in the pathogenesis of the disease. Oxidative stress causes both direct and indirect damage of germ cells, negatively influencing Sertoli cells and resulting in dramatic morphological alterations of the seminiferous epithelium and apoptosis induction [39]. In the present study, varicocele was reproduced in experimental animals by partial ligation of the left renal vein [3]. Varicocele rats showed a thin and atrophic epithelium with disorganized germ cells, reduced mean diameter of the tubules, and a low Johnsen’s score, as previously described [3,7,8,24,25,40,41,42]. These alterations were also slightly present in the contralateral testes. The modulation of the oxidative stress processes, thanks to lycopene use, significantly ameliorated structural damage of tubules in testes [43]. In our experiment, administration of lycopene, at a dose comparable to that previously utilized in experimental models of prostate growth induced in rats by testosterone [9], caused significant testicular protective effects, with an improvement of testes structure and an increase in both the diameter of the seminiferous epithelium and Johnsen’s score, thus indicating a possible improvement in male fertility.

Oxidative stress triggers apoptosis, which is considered one of the mechanisms that contribute to the pathophysiology of varicocele [44]. As a matter of fact, apoptotic activation was also observed in germ cells, in testicular tissues, and in ejaculated spermatozoa [45]; this impaired condition led us to hypothesize the presence of damaged DNA in sperm [46].

Varicocele-induced damage markedly increased and decreased the pro-apoptotic BAX and the anti-apoptotic Bcl-2 gene expression, respectively; this strongly suggests that the apoptotic process was stimulated in varicocele rats. Moreover, TUNEL-positive cells in the seminiferous tubules of varicocele testes were present, and the number of TUNEL-positive cells was increased compared to the sham rats. Since testes are constituted by a large amount of polyunsaturated fatty acids, and, at the same time, sperm cytoplasm contains low concentrations of scavenging enzymes, sperm cells are particularly susceptible to ROS; the elevated apoptotic cell rates observed in the present study let us hypothesize that apoptotic activation was related to increased ROS formation in testicular tissue.

Lycopene is involved in the scavenging of two of the reactive oxygen species (ROS): singlet molecular oxygen and peroxyl radicals [47]. Previous studies demonstrated that lycopene protected testes, thus increasing sperm motility and reducing apoptosis [48]. Interestingly, lycopene administration not only significantly reduced BAX and increased Bcl-2 expression, but also decreased the number of TUNEL-positive cells in the seminiferous tubules, demonstrating that lycopene significantly modulated and reduced apoptotic process. Then, these results confirm the potentially beneficial therapeutic effects of lycopene/lycopenoids, including the inhibition of carcinogenic activation, proliferation, angiogenesis, invasion and metastasis, and the blocking of tumor cell-cycle progression [49].

Hormonal dysfunction was observed in varicocele, and is involved in the pathophysiology of the disease, being closely linked to oxidative stress and apoptosis [1,2,41,45]. Indeed, spermatogenesis is a testosterone-dependent process, and hormonal imbalance can be a cause of impairment of spermatogenesis. In this regard, many authors have demonstrated reduced serum testosterone levels in men with varicocele [50], probably also as a consequence of oxidative stress production, and have provided findings supporting the repair of varicocele as resulting in the expected improvement of Leydig cell functions [45,46]. The model used in this experimental setting, based on a surgically induced varicocele, caused the reduction of circulating testosterone, probably also as a consequence of oxidative stress production, thus confirming the results obtained in previous studies that described the decrease of testosterone levels as being associated with an impaired function of the Leydig cells [51].

Testosterone is fundamental for spermatogenesis, and the weight of the testes is related to the quantity of spermatogenic cells and spermatozoa [52,53]. In fact, in the present study the decrease of the weight of testes observed in varicocele rats let us hypothesize that it was a consequence of the reduction of testosterone levels.

Lycopene administration significantly increased testosterone levels in varicocele-treated rats, thus indicating the positive role of lycopene in restoring the hormonal dysfunction and consequent infertility by experimentally induced varicocele. As a matter of fact, our experimental observations confirm the crucial role of steroid hormones and molecular signaling systems (i.e., insulin-like growth factor), giving new insight into the biological action of carotenoids, particularly lycopene [54,55,56,57]. Of course, in light of previous reports indicating that lycopenoids influence androgen metabolism in rodent models [14], additional experiments are required to better define the role of lycopene metabolites and their nutraceutical bioactivity.

## 5. Conclusions

In conclusion, taken together, our data suggest that lycopene use might be considered a novel strategy for the treatment of varicocele. However, additional and translational studies are required to study a new possible mechanism of action of this bioactive compound, typically present in the traditional MD, and the amount of dietary intake needed to carry out its curative effects in the management of varicocele and male infertility.

## Figures and Tables

**Table 1:** Effects on testis weight and testosterone induced by lycopene in varicocele rats as compared to varicocele and sham rats. All values are expressed as mean ± SD; *n* = 7 animals for each group.

	Testis Weight (g)	Testosterone (ng/mL)
Sham	1.552 ± 0.129	5.8 ± 0.7
Varicocele operated testis	0.926 ± 0.186 ^a^	2.6 ± 0.3 ^a^
Varicocele contralateral testis	1.608 ± 0.204 ^b^	
Varicocele + lycopene operated testis	1.386 ± 0.149 ^b^	5.2 ± 0.6 ^b^
Varicocele + lycopene contralateral testis	1.727 ± 0.223 ^b^	

^a^ = *p* < 0.05 vs. sham; ^b^ = *p* < 0.05 vs. varicocele.

**Table 2:** Effects on testis malondialdehyde induced by lycopene in varicocele rats as compared to varicocele and sham rats. All values are expressed as mean ± SD; *n* = 7 animals for each group.

	Malondialdehyde (μmol/mg Tissue)
Sham	2.12 ± 0.27
Varicocele operated testis	4.46 ± 0.45 ^a^
Varicocele contralateral testis	2.91 ± 0.38 ^b^
Varicocele + lycopene operated testis	2.37 ± 0.35 ^b^
Varicocele + lycopene contralateral testis	2.09 ± 0.29 ^b^

^a^ = *p* < 0.05 vs. sham; ^b^ = *p* < 0.05 vs. varicocele.
